# The natural course of idiopathic cervical dystonia

**DOI:** 10.1007/s00702-023-02736-0

**Published:** 2024-01-20

**Authors:** Dirk Dressler, Bruno Kopp, Lizhen Pan, Fereshte Adib Saberi

**Affiliations:** 1 Movement Disorders Section, Department of Neurology, Hannover Medical School, Carl-Neuberg-Str. 1, 30625 Hannover, Germany; 2 Department of Neurology, Hannover Medical School, Hannover, Germany; 3 Department of Neurology, Neurotoxin Research Center, Tongji University School of Medicine, Shanghai, China; 4 IAB-Interdisciplinary Working Group for Movement Disorders, Hamburg, Germany

**Keywords:** Idiopathic cervical dystonia, Natural course, Type 1, Type 2, Excessive psychological stress

## Abstract

Idiopathic cervical dystonia (ICD) is by far the largest subgroup of dystonia. Still, its natural course is largely unknown. We studied the natural course of 100 ICD patients from our botulinum toxin clinics (age at ICD onset 45.8 ± 13.5 years, female/male ratio 2.0) over a period of 17.5 ± 11.5 years with follow-ups during botulinum toxin therapy and with semi-structured interviews. Two courses of ICD could be distinguished by symptom development of more or less than 6 months. ICD-type 2 was less frequent (19% vs 81%, *p* < 0.001), had a more rapid onset (8.7 ± 8.0 weeks vs 3.8 ± 3.5 years), a higher remission rate (92% vs 5%, *p* < 0.001) and a higher prevalence of excessive psychological stress preceding ICD (63% vs 1%, *p* < 0.001). In both ICD-types, the plateau phase was non-progressive. Significant differences in patient age at ICD onset, latency and extent of remission, female/male ratio and prevalence of family history of dystonia could not be detected. ICD is a non-progressive disorder. ICD-type 1 represents the standard course. ICD-type 2 features rapid onset, preceding excessive psychological stress and a high remission rate. These findings will improve prognosis, treatment strategies and understanding of underlying disease mechanisms. They contradict the widespread fear of patients of a constant and continued decline of their condition. Excessive psychological stress may be an epigenetic factor triggering the manifestation of genetically predetermined dystonia.

## Introduction

The course of a disease is important for physicians to plan therapies, for patients to anticipate their prognosis and for basic scientists to understand the underlying disease mechanisms.

Idiopathic dystonia is defined as dystonia occurring in the absence of identifiable inherited or acquired causes and in the absence of a psychogenic aetiology (Albanese et al. 2013). Idiopathic cervical dystonia (ICD) is the largest subgroup of dystonia (Dressler et al. 2022). For special genetic dystonias, such as DYT1 dystonia (Oppenheim dystonia), early onset progressive courses have been described (Klein and Muenchau 2013). However, only few patients fall in this category. For ICD, the natural course has not been described in detail (Pana and Saggu 2021, Van Zandijcke 1995). As dystonia is generally considered a neurodegenerative disorder, its course is usually perceived as being progressive, similar to Parkinson's disease and Alzheimer's dementia. This means for physicians, that treatment strategies may need to be escalated. For basic scientists, it means that certain underlying disease mechanisms may be assumed and others may be discarded. Most importantly, this means for patients, that they live under the fear of a constant decline of their condition, both with respect to severity and with respect to spread to other body parts.

This study wants to describe the natural course of ICD. Results presented here will considerably change the conventional perception of ICD being a progressive disorder.

## Methods

### Design

The study is based on regular follow-ups complemented by semi-structured interviews of ICD patients. Most of them received regular botulinum toxin therapy at the Movement Disorders Section of the Department of Neurology of Hannover Medical School in Hannover, Germany.

### Patients

Patient inclusion criteria consisted of (1) diagnosis of ICD (2) Existence of ICD for at least one year (3) Willingness and ability to participate in the study (4) Regular follow-ups. Exclusion criteria included symptomatic cervical dystonia, psychogenic cervical dystonia and non-dystonic torticollis. Patients were seen in our botulinum toxin clinics and included consecutively into this study until the pre-set number of 100 patients was reached.

### Study parameters

The study parameters are shown in Table 1. Patient demographics described the patient's sex, the patient's age at study inclusion and the patient's age at ICD onset. The patient's family history of dystonia derived from personal examination of family members, diagnoses by other physicians and signs and symptoms provided by the patient. The level of diagnostic accuracy may be best described as 'probable'.

**Table 1:** Study parameters with their definitions and dimensions

Parameter	Definition	Dimension
Patient age	Patient's age at time of inclusion in this study	Years
Patient age at onset	Patient's age at time of ICD onset	Years
Patient sex	Patient's sex	Male/female
ICD course	Description of the patient's ICD with respect to severity over time	% of maximal severity/time
Time of maximal ICD severity	Time when ICD reached its maximal severity	Years/weeks after ICD onset
Onset phase	Time between ICD onset and time of maximal ICD severity	Years/weeks
Plateau phase	Time between maximal ICD severity and onset of remission phase	Years
Remission phase	Time between onset and end of remission	Years
Remission extent	Extent of ICD remission	% of maximal symptomatology
Observation period	Time between ICD onset and last patient contact	Years
Efficacy of BT therapy	Overall efficacy of BT therapy as estimated by the patient, including head posture and muscle pain	% of untreated condition
Duration of BT therapy	Time between BT therapy onset and end of observation period	years
Family history of dystonia	Patient's family history of dystonia	Yes/no (description)
Excessive psychological stress	Patient's exposure to stress with severity not been experienced before or thereafter	Yes/no (description)

*BT* botulinum toxin, *ICD* cervical dystonia

**Fig. 1:**
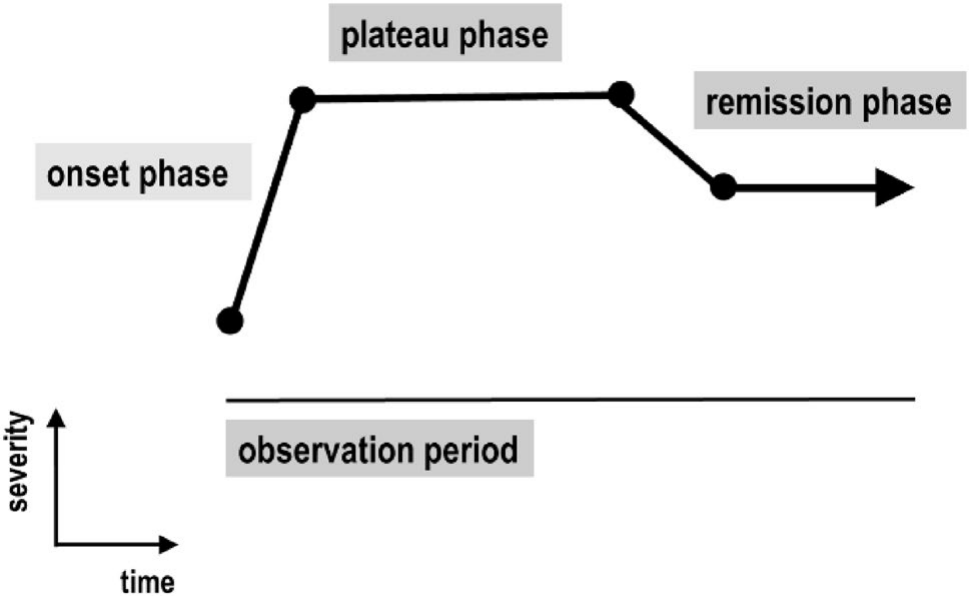
Schematic time course of idiopathic cervical dystonia. Explanation in text

The course of ICD is described by its onset phase, its plateau phase and its remission phase, as shown in Fig. 1. The observation period was defined as the time between ICD onset and last contact with the patient. In a semi-structured interview, the patient is shown Fig. 1 together with definitions of the onset phase, the plateau phase and the remission phase. Explanations were given in plain German language and the patients were allowed to ask clarifying questions until they and the interviewer thought the interview requests were understood. Based on the patient's information, the patient's individual ICD course was constructed.

Exceptional psychological stress was defined as psychological stress of a severity the patient had never experienced before or thereafter.

### Follow-ups

Neurological follow-ups were mostly performed during the continued botulinum toxin injection series usually scheduled at intervals of 3 months.

### Statistics

Study parameters were transferred into a matrix and correlations were calculated wherever they were of interest. The statistical tests applied are given in the text. All averages are given as mean ± standard deviation. Statistical significance was set to *p* ≤ 0.05.

## Results

### Patients

Altogether 100 consecutive ICD patients were included in this study. There were no dropouts. The patient age at ICD onset was 45.8 ± 13.5 years. The patient age at study inclusion was not used for further evaluations, as it may be confounded by the presence or absence of remissions. 66% of the patients were female, 34% were male, i.e. the female/male ratio was 2.0. The observation period was 17.5 ± 11.5 years.

### ICD course

After preliminary screening of the onset phase duration, patients were divided into two distinct groups as shown in Table 2. Separation was based on an onset phase of more or less than 6 months.

**Table 2:** Comparison of patients with ICD-type 1 (regular onset) and ICD-type 2 (rapid onset)

Feature	ICD-type 1 Regular onset	ICD-type 2 Rapid onset	Comparison ICD-type 1 and ICD-type 2	
Prevalence in all ICD patients	81%	19%	binomial test (H_0_: *p* = 0.5), *p* < 0.001	*
Patient age at ICD onset	46.9 ± 13.4y	41.5 ± 12.7y	Student's t-test *t (*1.58); *p* = 0.12	ns
Female/male ratio	2.00	1.71	Pearson's chi-squared test *X*^2^ = 0.08, *p* = 0.77	ns
Prevalence of family history of dystonia	28%	16%	Pearson's chi-squared test *X*^2^ = 1.27, *p* = 0.26	ns
Prevalence of excessive psychological stress	1%	63%	Pearson's chi-squared test *X*^*2*=^*52.22; p* < 0.001	*
Duration of onset phase	3.8 ± 3.5y	8.7 ± 8.0w	n/a	
Prevalence of patients with remissions	5%	63%	Pearson's chi-squared test *X*^*2*^ = *38.81; p* < 0.001	*
Onset of remission	14.3 ± 8.3y	1.2 ± 0.4y	nsa	
Extent of remission	22.5 ± 18.9%	52.5 ± 34.4%	nsa	
Prevalence of patients with additional dystonic manifestations before ICD onset	11%	1%	nsa	
Prevalence of patients with additional dystonic manifestations after ICD onset	26%	16%	nsa	

*n/a* not applicable, *ns* not significant, *nsa* no statistical analysis due to small sample sizes, *w* weeks, *y* years, *ICD* cervical dystonia

*Statistical significance

#### ICD-type 1 (regular onset)

**Fig. 2:**
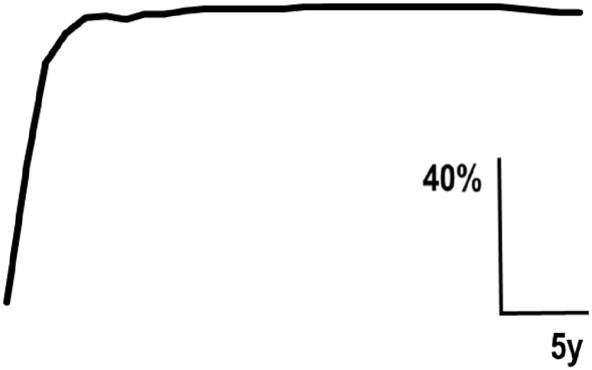
The natural course of cervical dystonia-type 1 as reconstructed from data from 81 patients

81% of all ICD patients belonged to this group. 67% of them were female, 33% male, i.e. their female/male ratio was 2.0. Their age at ICD onset was 46.9 ± 13.4 years. 28% had a family history of dystonia. One patient (1%) had experienced excessive psychological stress preceding ICD onset. The natural course of all ICD-type 1 patients is shown in Fig. 2. The onset phase was 3.8 ± 3.5 years (minimum 1 year, maximum 30 years). Their plateau phase was 14.9 ± 10.3 years. During this time, there was no further disease progression. In 5% of these patients, there was remission. It started 14.3 ± 8.3 years after ICD onset and had an extent of 22.5 ± 18.9% (minimum 10%, maximum 50%) of the maximal severity. All remissions (with one exception) occurred after the patient's retirement. 11% of ICD-type 1 patients had mild additional dystonia manifestations before ICD onset, including blepharospasm (6%), oromandibular dystonia (4%), writer's cramp (3%) and arm dystonia (3%). 26% of ICD-type 1 patients had mild additional dystonia manifestations after ICD onset including arm dystonia (9%), oromandibular dystonia (7%), spasmodic dysphonia (6%), blepharospasm (6%), writer's cramp (4%) and axial dystonia (3%). The observation period in ICD-type 1 was 19.1 ± 11.7 years.

#### ICD-type 2 (rapid onset)

**Fig. 3:**
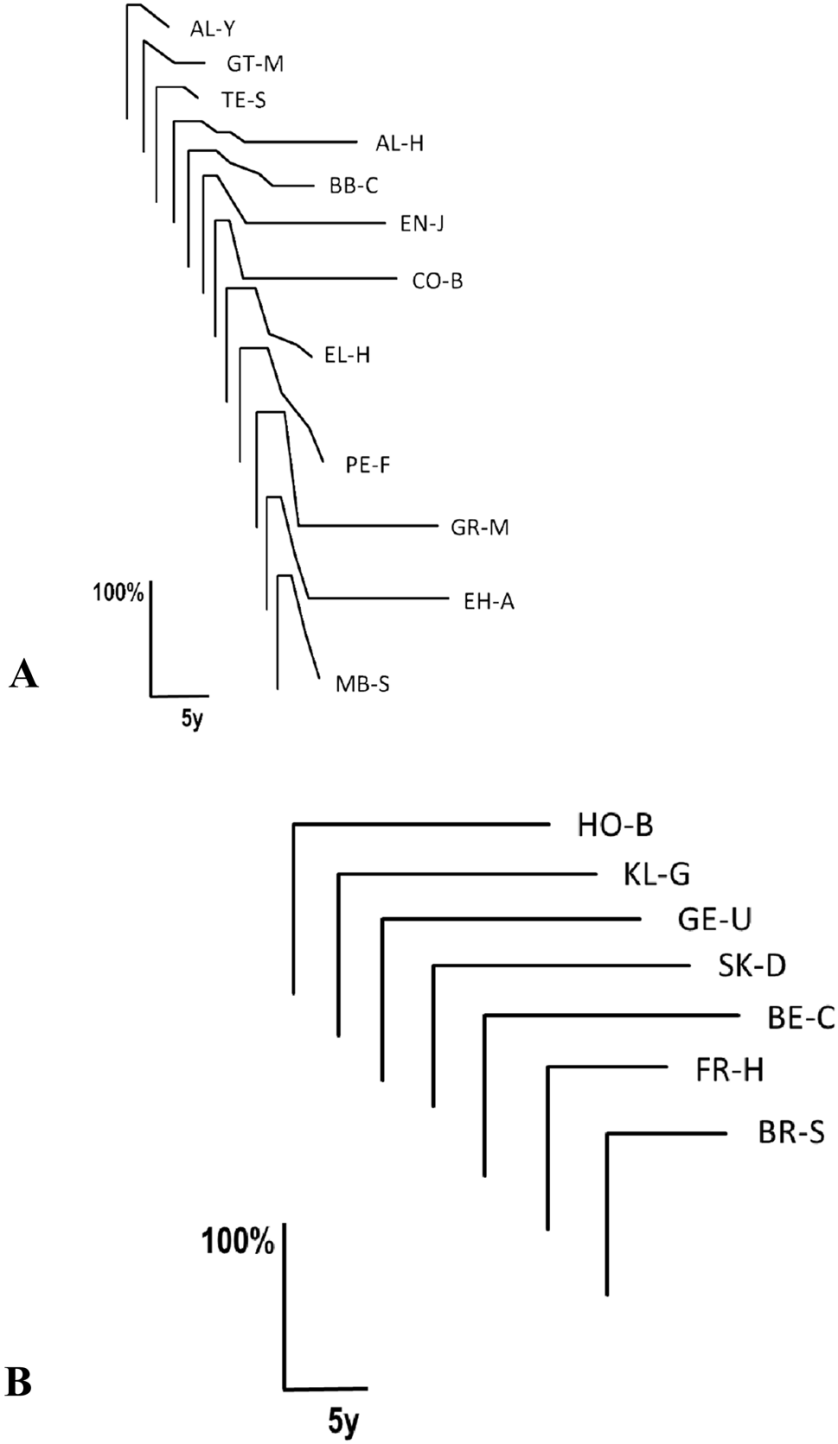
The natural course of idiopathic cervical dystonia type 2 in each individual patient. **A** Patients with remissions. **B** Patients without remissions

19% of all ICD patients belonged to this group. Their age at ICD onset was 41.5 ± 12.7 years. 63% of ICD-type 2 patients experienced excessive psychological stress. 16% of them had a family history of dystonia, 63% of them were female, 37% male, i.e. the female/male ratio was 1.7. The natural course of each ICD-type 2 patient is shown in Fig. 3. The onset phase of ICD-type 2 patients was 8.7 ± 8.0 weeks (minimum 1 week, maximum of 24 weeks by definition). 63% of ICD-type 2 patients experienced remissions. They started 1.2 ± 0.4 years after ICD onset with an extent of 52.5 ± 34.4% (minimum 10%, maximum 100%). As in ICD-type 1 patients, the plateau phase was never progressive. 16% of ICD-type 2 patients developed mild additional dystonia manifestations after ICD onset including arm dystonia, spasmodic dysphonia and blepharospasm (no statistical analysis due to small sample sizes). One % of ICD-type 2 patients developed mild additional dystonia manifestations before ICD onset (no statistical analysis due to small sample size). None of these additional manifestations dominated the clinical picture.

Compared to ICD-type 1, ICD-type 2 was less frequent (19% versus 81%, binomial test (H_0_: *p* = 0.5), *p* < 0.001), had more often excessive psychological stress preceding ICD onset (63% versus 1%, Pearson's chi-squared test, *Χ*^*2*^ = 52.22; *p* < 0.001), more remissions (63% versus 5%, Pearson's chi-squared test, *X*^*2*^ = 38.81; *p* < 0.001) and its onset phase was shorter (8.7 ± 8.0 weeks versus 3.8 ± 3.5 years. Whether latency and extent of the remissions and prevalence of patients with additional dystonic manifestations before or after ICD onset were different in both groups, could not be decided due to limited sample size in ICD-type 1. Age at ICD onset (41.5 ± 12.7 years versus 46.9 ± 13.4 years, Student's t-test, *t* (98) = 1.58; *p* = 0.12), frequency of family history with dystonia (16% versus 28%, Pearson's chi-squared test, *X*^*2*^ = 1.27; *p* = 0.26) and female/male ratio (1.7 versus 2.0, Pearson's chi-squared test, *X*^*2*^ = 0.08; *p* = 0.77) were not significantly different.

### Patients with excessive psychological stress

13% of all ICD patients experienced excessive psychological stress preceding ICD onset, including partner conflicts (divorce and separation, domestic violence), special familial burdens, legal disputes and migration. In patients with excessive psychological stress, age at ICD onset was 39.0 ± 13.9 years, onset phase 0.3 ± 0.8 years, remission rate 92%, remission extent 54.5 ± 35.3%, female/male ratio 1.2.

Compared to patients without excessive psychological stress, age at onset was lower (39.0 ± 13.9 years versus 46.9 ± 13.2 years, Student's t test, *t* (98) = 1.99; *p* = 0.05), onset phase was shorter (0.3 ± 0.8 years versus 3.6 ± 3.5 years, Student's t-test, *t* (98) = 3.31; *p* = 0.001) and the remission rate was higher (92% versus 5%, Pearson's chi-squared test, *Χ*^*2*^ = 52.34; *p* < 0.001). The female/male ratio was not different (1.2 versus 2.1, Pearson's chi-squared test, *Χ*^*2*^ = 0.98; *p* = 0.32).

### Patients with remissions

16% of all ICD patients experienced remissions. 75% patients with remissions had experienced excessive psychological stress, whereas only 2% of patients without remissions experienced exceptional psychological stress (Pearson's chi-squared test, *Χ*^*2*^ = 52.34; *p* < 0.001). The presence of remissions was correlated with lower patient age (55.9 ± 14.8 years vs. 65.1 ± 11.8 years, Student's *t* test, *t* (98) = 2.70; *p* < 0.01) and male patient sex (female/male ratio 0.8 versus 2.4, Pearson's chi-squared test, *Χ*^*2*^ = 4.20; *p* < 0.05), but not with patient age at ICD onset (versus, Student's *t* test, *t* (98) = 1.62; *p* = 0.11) or the presence of a family history of dystonia (23% versus 36%, Pearson's chi-squared test, *Χ*^*2*^ = 0.52; *p* = 0.47).

### Family history of dystonia

26% of all ICD patients had a family history of dystonia. Table 3 shows the various dystonia manifestations and their frequencies in 42 family members with history of dystonia. A family history of dystonia was not correlated with patient age at ICD onset (41.7 ± 16.8 years 47.3 11.9 years, Student's *t* test, *t* (98) = 1.96; *p* = 0.07), patient sex (female/male ratio 2.0 versus 1.9, Pearson's chi-squared test *Χ*^*2*^ = 0.01; *p* = 0.94), ICD-type 2 (3/26 12% versus 16/74, Pearson's chi-squared test, *Χ*^*2*^ = 1.27; *p* = 0.26) or the occurrence of remissions (13% versus 22%, Pearson's chi-squared test, *Χ*^*2*^ = 0.52; *p* = 0.47). In families with history of dystonia, an average of 1.8 ± 1.2 family members (minimum 1, maximum 5) were affected. Table 3 shows their dystonia manifestations and their relative frequencies.

**Table 3:** Dystonia manifestations and their frequencies in 42 family members with history of dystonia

Dystonia manifestation	Frequency (%)
Arm tremor	26
Writer's cramp	21
Head tremor	17
Cervical dystonia	14
Blepharospasm	12
Cervical dystonia together with blepharospasm	5
Spasmodic dysphonia	2

### Botulinum toxin therapy

97% of all patients in this study received botulinum toxin therapy. The duration of botulinum toxin therapy was 11.5 ± 9.8 years (minimum 1 year, maximum 41 years). Efficacy of botulinum toxin therapy was a subjective improvement of 73.2 ± 13.9% (minimum 30%, maximum 90%). No patient developed antibody-induced therapy failure and no patient terminated botulinum toxin therapy.

## Discussion

### General observations

With 100 patients included and an observation period of 17.5 ± 11.5 years, this study is—to our knowledge—the most detailed study on the natural course of ICD.

### Patients

ICD patients reported here, confirm previous demographical features with a patient age of 63.6 ± 12.7 years, an age of ICD onset of 45.8 ± 13.5 years and a strong female sex preponderance of 2.0.

### Botulinum toxin therapy

Botulinum toxin therapy is used in 98% of our patients. The subjective therapy effect indicates an improvement of 73.2 ± 13.9% including dystonic head movements as well as pain. During an observation period of 11.5 ± 9.8 years, we have not seen antibody-induced therapy failure, nor have we seen therapy termination. However, as this was not a prospective study, we have to refrain from definite statements.

### Family history of dystonia

26% of all ICD patients had a family history of dystonia with a wide spectrum of dystonia manifestations, often different from the dystonia manifestation of the index case. This confirms, that dystonia manifestations within the families may vary considerably. As most of the family members could not be examined personally, the diagnosis had to be described as 'probable'. It is noteworthy, that reports on the family history dating back to more than one generation are usually not available. Currently, cases of tremor without other dystonic manifestation would not be diagnosed as dystonic tremor. Our study suggests, not to consider clinical findings alone, but also to consider the family history in patients with tremor. In the future, classification systems of tremor should allow considering tremors without other dystonic manifestation as dystonic, when the patients family history is positive for dystonia. High numbers of family members affected by tremor and a substantial proportion of patients with presumed essential tremor actually suffering from dystonia support this view. A family history of dystonia was not correlated with patient age, age at ICD onset, patient sex, rapid dystonia onset and remissions.

### ICD course

The natural course of ICD can be divided into two distinct types with the very different features shown in Table 2.

#### ICD-type 1 (regular onset)

With 81% of all ICD patients, this type covers the standard situation. ICD-type 1 develops over a period of 3.8 ± 3.5 years. Thereafter, its course is constant. Further progression would be uncommon, but might occur in special rare cases. In 26% of patients, mild additional dystonic manifestations may occur after ICD onset. Remissions are very rare, are mild to moderate at best and occur late, often when patients retire. This course resembles the course previously described (Meares 1971). It remains unclear, whether these remissions reflect actual improvement of the condition or increased resilience, because of improved resources after retirement. With a gradual onset over about 4 years and a constant course, ICD-type 1 is a non-progressive disorder clearly different from other neurodegenerative conditions such as Parkinson's disease and Alzheimer's dementia.

#### ICD-type 2 (rapid onset)

This type covers 19% of all ICD patients. ICD-type 2 develops rapidly within 8.7 ± 8.0 weeks. 63% of these patients experienced excessive psychological stress preceding disease onset. For patients, this situation seems dramatic with its rapid onset and its often substantial disease severity. However, about two thirds of patients will experience substantial remissions within 1.2 ± 0.4 years. These remissions might be reflected in previous reports of ICD remissions (Bräutigam 1954; Jayne et al. 1984; Friedman & Fahn 1986). Very rarely, patients may experience repeated episodes of ICD-type 2. Patient age at ICD onset, patient sex, family history of dystonia and additional dystonia manifestations before or after ICD onset are not different between both types. The decisive difference between both ICD-types is preceding excessive psychological stress. Rapid disease onset in our patients with ICD and preceding excessive psychological stress should not be confused with abrupt disease onset in patients with psychological or functional disease.

### Differential diagnosis of rapid onset torticollis

Rapid onset neck spasms, generally known as torticollis, are seen in the conditions featured in Table 4. Most commonly, rapid onset neck spasms may be caused by non-dystonic mechanisms, typically by mechanical irritation of the peripheral neuromusculoskeletal system (Maigne et al. 2003). These cases of non-dystonic torticollis are typically tonic and painful. They recover quickly, especially under analgesics and muscle relaxants.

**Table 4:** Causes for rapid onset neck spasms

Entity	Remarks
Non-dystonic torticollis	Very common, spontaneous remissions, triggering event with pain
Psychogenic dystonia	Often fluctuations, additional symptoms, distractibility
Dystonia due to CNS lesions	Extremely rare, static course
Neuroleptic induced dystonia	Typically oromandibular-lingual manifestation, young males, prompt response to anticholinergics
ICD-type 2 triggered by exceptional stress	Often severe, high chance of remission
Shell shock dystonia	Mixed symptoms

*ICD* cervical dystonia

If rapid onset torticollis is caused by dystonia, it is probably most frequently caused by application of dopamine receptor blocking agents. Typically, young men may produce oromandibular lingual dystonia immediately after application of classical neuroleptics. ICD may also be part of such an acute neuroleptic reaction. Prompt response to anticholinergics is a hallmark of this condition. Rapid onset torticollis of dystonic origin may also be caused by sudden central nervous system pathologies such as stroke (LeDoux and Brady 2003). These cases are extremely rare and usually have a static course.

Recently, psychogenic or functional dystonia was defined as a manifestation of dystonia, rather than pseudo-dystonia as before. Abrupt onset—often in conflict situations—is a hallmark of psychogenic or functional movement disorders (Hallett 2016). Abrupt onset in psychogenic movement disorders is typically within hours or days, thus different from our ICD-type 2 patients with rapid, but not abrupt onset. Valid data about prevalence, natural course and treatment of psychogenic ICD are still lacking. A recent study suggests a prevalence of psychogenic dystonia of 28.5/1mio or of 5% of all dystonia patients (Dressler et al. 2022). Within psychogenic dystonia, psychogenic ICD is relatively common. In our study, non-dystonic torticollis, drug-induced ICD, other symptomatic ICD and psychogenic ICD were explicitly excluded.

### Excessive psychological stress

13% of all ICD patients experienced excessive psychological stress preceding ICD onset. In 85% of these patients, ICD developed within 5.8 ± 4.4 weeks, then lasted 18.5 ± 8.3 months, before it started to remit after 2.7 ± 0.8 years to 54.5 ± 35.3% of its maximal severity. ICD with preceding excessive psychological stress is closely connected with ICD-type 2: 63% of patients with ICD-type 2 experienced preceding excessive psychological stress and 84% of patients with preceding excessive psychological stress presented with ICD type 2. It is challenging, to elucidate the interactions between ICD and excessive psychological stress. This was performed in another publication of ours (Dressler et al. 2023).

### Patient counselling

Our data clearly show, that both ICD-types are non-progressive. For patient counselling, we assume no major further deterioration, once ICD has been stable for two or three years. This is important information for patients, as most of them intuitively assume a chronic progressive course similar to other neurodegenerative disorders such as Parkinson's disease or Alzheimer disease.

## Outlook

This is the first description of two distinct types of ICD. For both types, the natural course is non-progressive. This distinction should be included in the classification of ICD, as it has immediate relevance for the counselling and treatment of our patients and for the basic scientist's understanding of the pathological mechanisms underlying ICD.

Further studies will have to show, whether the findings described here for ICD will also apply to other focal and non-focal dystonias.

## Data Availability

All data are on file and may be reviewed upon reasonable request.
